# Transcriptomic Analysis of Coding Genes and Non-Coding RNAs Reveals Complex Regulatory Networks Underlying the Black Back and White Belly Coat Phenotype in Chinese Wuzhishan Pigs

**DOI:** 10.3390/genes10030201

**Published:** 2019-03-07

**Authors:** Qiao Xu, Ximing Liu, Zhe Chao, Kejun Wang, Jue Wang, Qiguo Tang, Yabiao Luo, Jie Zheng, Shuyi Tan, Meiying Fang

**Affiliations:** 1 Department of Animal Genetics and Breeding, National Engineering Laboratory for Animal Breeding, MOA Laboratory of Animal Genetics and Breeding, Beijing key Laboratory for Animal Genetic Improvement, College of Animal Science and Technology, China Agricultural University, Beijing 100193, China; xuqiao987@163.com (Q.X.); Lximing2018@163.com (X.L.); wangkejun.me@163.com (K.W.); tctcttc@hotmail.com (J.W.); tango@cau.edu.cn (Q.T.); flynluo@163.com (Y.L.); Katharinezj@163.com (J.Z.); 2 Institute of Animal Sciences and Veterinary, Hainan Academy of Agricultural Sciences, Haikou 571100, China; chaozhe.cn@126.com (Z.C.); tanshuyi1168@sina.com (S.T.)

**Keywords:** Chinese Wuzhishan pig, black back, white belly, coat color, lncRNA–miRNA–gene

## Abstract

Coat color is one of the most important characteristics for distinguishing Chinese indigenous pig breeds. In Wuzhishan pigs, the animals have black on the back and white on the abdomen. However, the molecular genetic basis of this phenotype is unclear. In this study, we used high-throughput RNA sequencing to compare expression profiles of coding and non-coding RNAs from white and black skin samples obtained from individual Wuzhishan pigs. The expression profiling revealed that 194 lncRNAs (long non-coding RNAs), 189 mRNAs (messenger RNAs), and 162 miRNAs (microRNAs) had significantly different levels of expression (|log_2_ fold change| > 1, *p*-value < 0.05) in white and black skin. Compared to RNA levels in black skin, white skin had higher levels of expression of 185 lncRNAs, 181 mRNAs, and 23 miRNAs and lower levels of expression of 9 lncRNAs, 8 mRNAs, and 139 miRNAs. Functional analysis suggested that the differentially expressed transcripts are involved in biological processes such as melanin biosynthesis, pigmentation and tyrosine metabolism. Several key genes involved in melanogenesis, including *MLANA*, *PMEL*, *TYR*, *TYRP1*, *DTC*, *TRPM1* and *CAMK2A,* had significantly different levels of expression in the two skin tissues. Potential lncRNA–miRNA–gene interactions were also examined. A total of 15 lncRNAs, 11 miRNAs and 7 genes formed 23 lncRNA–miRNA–gene pairs, suggesting that complex regulatory networks of coding and non-coding genes underlie the coat color trait in Wuzhishan pigs. Our study provides a foundation for understanding how lncRNA, miRNA and genes interact to regulate coat color in black-back/white-belly pigs. We also constructed lncRNA–miRNA–gene interaction networks to elucidate the complex molecular mechanisms underlying skin physiology and melanogenesis. The results extend our knowledge about the diversity of coat color among different domestic animals and provide a foundation for studying novel mechanisms that control coat color in Chinese indigenous pigs.

## 1. Introduction

Pig coat color was the focus of pioneering genetic studies carried out at the beginning of last century [1,2]. In general, while wild boars are relatively uniformly colored, a wide variety of coat colors are found in domestic pigs. The shift from natural selection towards criteria based on human preferences accounts for most of the increase in coat color variation. Coat color is readily observable and can be used to distinguish different species, breeds or individuals. Thus, it has long been a subject of fascination. However, the underlying genetic mechanisms of pigmentation are often difficult to decipher.

Coat color diversity results from the presence and biochemical activity of melanocytes, which are specialized in producing melanins [3]. Melanins are divided into eumelanins (black/brown, pigmented phenotype) and pheomelanins (red/yellow, non-pigmented phenotype). These two pigments are sufficient in producing the wide variety of coat colors observed in all mammals. Genes regulating skin and coat color can be grouped into two categories: one regulates the production, proliferation or migration of different types of melanocytes and the other directly affects pigment synthesis. Therefore, the formation of different skin and coat colors is determined by the regulation of genes, which can change the progression/differentiation of melanocytes or the process of melanin synthesis. The molecular genetic mechanism of coat color variation has previously been investigated in pigs [4,5] and many of the genes that control coat color in this animal also regulate coat color in other species [6,7,8,9]. 

Transcriptional profiling is a powerful approach for identifying genes and their expression patterns in tissues, including skin [10,11,12,13,14], hair follicles [15,16,17] and feather bulbs [18,19,20]. Profiles can include both coding RNAs and non-coding RNAs (ncRNAs), such as micro RNAs (miRNAs) and long non-coding RNAs (lncRNAs). Most expression profiles generated to study skin or coat color have been obtained from sheep [10], horses [14], chickens [21] and mink [13]. There is growing evidence that miRNA plays an important role in regulating the expression of skin and hair follicle genes at the post-transcriptional level [17,22,23]. lncRNAs are a class of regulatory ncRNAs with sizes ranging from 200 bp to 100 kb [24]. Researchers have identified several lncRNAs associated with skin biology, such as *ANCR*, *TINCR*, *U1 RNA*, *PRINS*, *BANCR* and *SPRY4-IT1* [25]. However, only a few skin-associated lncRNAs are known to be involved in determining coat color in cattle [26] and sheep [27] and very few miRNAs and lncRNAs related to coat color differences in pigs have been described. Most studies have mainly focused on polymorphisms of candidate genes (review in [5]), while limited information is available on how coat color phenotypes may be regulated at the transcriptional level, especially in Chinese indigenous breeds, such as Lantang and Wuzhishan pigs, which have black backs and white abdomens.

In this study, our aim is to use RNA-sequencing (RNA-seq) to investigate the transcriptional patterns for genes, lncRNAs and miRNAs that may function to regulate coat color in the black-back/white-belly Wuzhishan pig. The results provide insight into the complex molecular mechanisms underlying skin physiology and melanogenesis, which will also expand our understanding of coat color diversity in different domestic animals. 

## 2. Materials and Methods 

### 2.1. Animal Sample Collection and RNA Isolation

**Figure 1:**
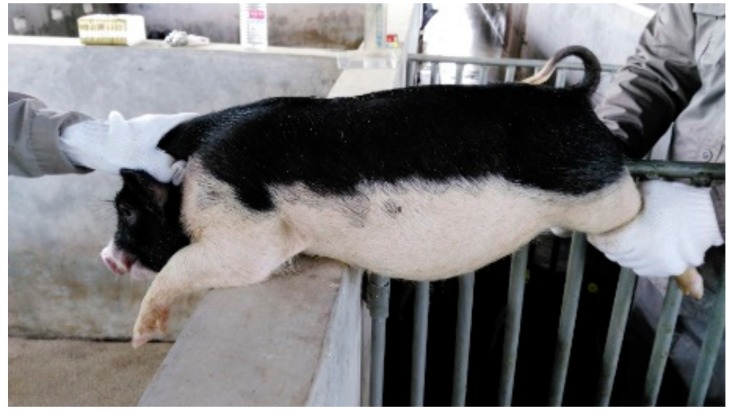
A Wuzhishan pig, showing the characteristic black back and white belly.

Three unrelated black-back/white-belly Wuzhishan pigs, which were raised under identical conditions, were randomly selected for the study. From each pig, a skin punch biopsy that had a diameter of 1 cm and a thickness of 4 cm was collected from the abdomen and the back. All procedures were done under local anesthesia at the Institute of Animal and Veterinary Sciences at the Hainan Academy of Agricultural Sciences (Haikou, China) (Figure 1). Samples were immediately frozen in liquid nitrogen and stored at −80 °C. The white and black skin were found to have similar cellular composition and the melanocytes were identified in white skin by DOPA (3,4-Dihydroxy-L-phenylalanine) staining (Figure S1). Animal care and experiments were conducted according to the Regulations for the Administration of Affairs Concerning Experimental Animals (Ministry of Science and Technology, China; revised June 2004) and were approved by the animal welfare committee of the State Key Laboratory for Agro-biotechnology of China Agricultural University (approval number XK257).

Total RNA was isolated using TRIZOL^®^ Reagent (Invitrogen, San Diego, CA, USA) according to the manufacturer’s instructions. RNA quality was assessed using 1% agarose gels. RNA purity was determined using a K5500 Spectrophotometer (Kaiao, Beijing, China). RNA integrity and concentration was assessed using the RNA Nano 6000 Assay Kit and the Bioanalyzer 2100 system (Agilent Technologies, Foster, CA, USA). 

### 2.2. Library Preparation for Long Non-Coding RNA Sequencing and Data Analysis

Sequencing libraries were prepared using 3 μg of RNA per sample. Samples were indexed with the NEBNext^®^ Ultra™ Directional RNA Library Prep Kit for Illumina (NEB, Ipswich, MA, USA), following the manufacturer’s recommended procedures. Briefly, ribosomal RNA was removed using an Epicentre Ribo-Zero™ Gold Kit (Epicentre, Madison, WI, USA). RNA was fragmented to generate short RNA strands using NEBNext First Strand Synthesis Reaction Buffer (NEB, Ispawich, MA, USA) at an elevated temperature. The first complementary DNA (cDNA) strand was synthesized using random hexamer primers and RNA fragments as the template. Second strand cDNA synthesis was performed in a reaction with buffer, deoxyribonucleotide triphosphates (dNTPs), DNA polymerase I and RNase H. Overhangs were converted into blunt ends by the exonuclease/polymerase activities. Library fragments were purified using a QiaQuick PCR kit (Qiagen, Chatsworth, CA, USA) and eluted in EB buffer, with termini being repaired. After this, poly(A) and adapters were added. In order to preferentially select cDNA fragments of ca. 300 bp in length, library fragments were purified by agarose gel electrophoresis and the UNG enzyme was used to digest the second cDNA strand. PCR was performed and products of the desired size were isolated by electrophoresis through a 1% agarose gel. Finally, products were purified using the AMPure XP system and library quality was assessed with an Agilent Bioanalyzer 2100 system (Agilent Technologies). Libraries were sequenced on a HiSeq 4000 platform (Illumina, San Diego, CA, USA) and 150 bp paired-end reads were generated. Raw data (raw reads) were processed using Perl scripts to remove reads containing an adapter, reads containing poly-N and reads of low-quality. A low-quality read was defined as one in which more than 15% of bases had Phred quality scores less than or equal to 19. The remaining reads were mapped to the porcine reference genome (Sscrofa 11.1, Ensembl, ftp://ftp.ensembl.org/pub/release-92/fasta/sus_scrofa/dna/) using HiSAT2 (http://ccb.jhu.edu/software/hisat2/index.shtml) [28]. Mapped reads from each skin sample were assembled using StringTie [29] in a reference-based approach. After this, we evaluated the assembled transcripts using five criteria to identify lncRNAs: (1) transcripts with exon number < 2 were removed; (2) transcripts with length ≤ 200 bp were removed; (3) known no-lncRNA annotations were removed; (4) transcripts with Fragments Per Kilobase of exon per Million fragments mapped (FPKM) < 0.5 were removed; (5) coding-non-coding-index (CNCI) v2 [30], coding potential calculator (CPC) 0.9-r2 [31], PFAM-scan v1.3 [32], and coding potential assessment tool (CPAT) [33] were used to distinguish mRNAs from lncRNAs. Transcripts predicted to have coding potential by all of the four tools described above were excluded and those without coding potential were classified as lncRNA candidates. The transcripts excluded above were used as candidate mRNAs. PHAST v1.3 [34] was used for conservation analysis for coding genes and lncRNAs [35].

DESeq v1.16 [36] was used for differential gene expression analysis of lncRNAs and mRNAs between white and black skin. LncRNAs and mRNAs with a *p*-value < 0.05 and | log_2_ fold change | > 1 are identified as being differentially expressed between two groups. We searched for potential cis targets (i.e., coding genes) 50 kb upstream and downstream from each lncRNA. Potential trans targets were identified by examining RNA data for coordinated expression, using Pearson’s correlation coefficients (*r* > 0.90 or *r* < −0.90) as a classifier.

### 2.3. Library Preparation for Micro RNA Sequencing and Data Analysis

Three μg of RNA per sample was used as the input material for the small RNA library. Sequencing libraries were generated using the NEBNext^®^ Multiplex Small RNA Library Prep Set for Illumina^®^ (NEB, Ispawich, MA, USA), following the manufacturer′s recommendations. Index codes were added to distinguish sequences from each sample. Briefly, NEB 3′ SR Adaptors were ligated to 3′ ends of miRNA, small interfering RNA (siRNA) and piwi-interacting RNA piRNA. After ligation, sequence read (SR) reverse transcription (RT) Primer was hybridized to the remaining free 3′ SR Adaptor, which converted the single-stranded DNA adaptor to a double-stranded DNA molecule. Following this, 5′ end adapters were ligated to the 5′ends of miRNAs, siRNA and piRNA. First strand cDNA was synthesized using M-MuLV Reverse Transcriptase (RNase H–). PCR amplification was performed using LongAmp Taq 2X Master Mix, SR Primer for Illumina and index (X) primer. PCR products were purified on an 8% polyacrylamide gel (100 V, 80 min). DNA fragments corresponding to 140~160 bp (the length of a small noncoding RNA plus the 3′ and 5′ adaptors) were recovered and dissolved in 8 μL of elution buffer. Finally, the library quality was assessed on the Agilent Bioanalyzer 2100 system using DNA High Sensitivity Chips. The clustering of the index-coded samples was performed on a cBot Cluster Generation System using the TruSeq SR Cluster Kit v3-cBot-HS (Illumina), according to the manufacturer’s instructions. After cluster generation, the library preparations were sequenced on an HiSeq 2500/2000 platform (Illumina) and 50 bp single-end reads were generated. Raw data (raw reads) were processed with Python scripts to remove defective reads, i.e., those containing poly-N with lengths outside of the acceptable range, missing the 3′ adapter or insert fragment, containing excessive poly A/T or having low quality scores (the number of Reads Bases whose Phred quality value was less than or equal to 19 accounted for more than 15%). Clean reads with lengths in the desired range were used in all downstream analyses. The small RNAs were mapped to the porcine reference genome (*Sus scrofa* 11.1) using Bowtie [37] (no mismatches permitted) to analyze expression and genomic distribution. Bedtools (https://bedtools.readthedocs.io/) was used to search for known miRNAs by matching them to entries in miRBase20.0 (http://www.mirbase.org/). After excluding reads that were mapped to known miRNAs, miRDeep2 [38] was used to analyze the remaining reads to predict novel miRNAs. The prediction of miRNA target genes was performed using miRanda [39]. The levels of miRNA expression were estimated by RPM (reads per million total reads). Differential expression of miRNA between samples was analyzed using DEGseq v1.18.0. miRNAs with significant differences (*p*-value < 0.05) and | log_2_ fold change | > 1 were classified as differentially expressed (DE) miRNA.

All RNA-seq data was deposited into the Sequence Read Archive (SRA) database from NCBI (https://www.ncbi.nlm.nih.gov/sra). The accession numbers of lncRNA are SRR8173548, SRR8173549, SRR8173552, SRR8173553, SRR8173554, SRR8173555. The accession numbers of micro RNA are SRR8173550, SRR8173551.

### 2.4. Gene Ontology and Kyoto Encyclopedia of Genes and Genomes Enrichment Analysis

Gene ontology enrichment analysis of differentially expressed genes (DEGs) or DE lncRNAs and DE miRNAs target genes was conducted using the GO-seq R package [40], correcting for gene length bias. GO terms with *p*-values less than 0.05 were considered to be significantly enriched by DEGs. We used KOBAS v3.0 (http://kobas.cbi.pku.edu.cn/) [41] to test the statistical significance for the enrichment of DEGs or targets of DE lncRNA and DE miRNAs in KEGG pathways.

### 2.5. Construction of LncRNA–miRNA–Gene Regulatory Networks

To construct the lncRNA–miRNA–target gene network, we first used BLASTN (https://blast.ncbi.nlm.nih.gov/) to identify and remove pre-microRNAs based on high levels of homology. Subsequently, miRanda was used to predict the target relationships between miRNAs and lncRNAs (we required an alignment score *N* = 160 and a minimum free energy of −20 kcal/mol). As a competing endogenous RNA (ceRNA), a lncRNA can competitively bind miRNA with mRNA. Therefore, lncRNA–miRNA–gene pairs were further analyzed based on the common miRNA-binding sites [42]. The lncRNA, miRNA and mRNA interactions were constructed and visualized using Cytoscape v3.2.1 [43].

### 2.6. Quantitative Polymerase Chain Reaction

For detecting DE lncRNAs and DEGs, the total RNA (1 μg) from skin was transcribed into cDNA using the Fast Quant RT Kit (with gDNase) (Tiangen Biotech Co., Ltd, Beijing, China), according to the manufacturer’s instructions. Detecting DE miRNAs, the total RNA (1 μg) from skin was transcribed into cDNA using the TaqMan^®^ MicroRNA Reverse Transcription Kit (Applied Biosystems, Foster City, CA, USA), according to the manufacturer’s instructions. Expression levels of six lncRNAs, six genes and six miRNAs were quantified with quantitative PCR (qPCR) using SYBR Green Real-time PCR Master Mix (Tiangen Biotech Co., Ltd, Beijing, China). Gene and lncRNA primers for qPCR were designed using Primer Premier 5.0 (Premier Biosoft International, Palo Alto, CA, USA) and were subsequently synthesized (Sangon Biotech, Beijing, China). MiRNA primers were designed and synthesized by RiboBio (Guangzhou, China). Target lncRNAs, genes and miRNA are listed in Table 1 while gene and lncRNA primer sequences are listed in Table S1. The cycling parameters used for qPCR amplification were as follows: initial heat denaturation at 95 °C for 15 min, 40 cycles at 95 °C for 30 s, 60 °C for 30 s and 72 °C for 30 s; and a final extension at 72 °C for 5 min. A melting curve analysis was performed to exclude genomic DNA contamination and to confirm primer specificities. Gene and lncRNA expression levels were normalized using the 2^−ΔΔCt^ method with *GAPDH* (the expression of *GAPDH* was identified as stable in skin samples by semi-quantitative reverse transcription (RT)-PCR) as an internal standard. Relative miRNA expression was normalized using the 2^−ΔΔCt^ method with the U6 small nuclear RNA as an internal standard. Each biological duplicate consisted of three technical replicates.

### 2.7. Statistical Analysis

Data were expressed as means ± standard deviation (SD). Significance was analyzed using one-way analysis of variance (ANOVA) to test homogeneity of variances via Levene’s test, followed by Student’s *t*-test. Calculations were conducted using SAS version 9.0 (SAS, Cary, NC, USA). Differences were considered to be statistically significant for *p*-values < 0.05.

## 3. Results

### 3.1. Identification of Long Non-Coding RNAs, Messenger RNAs and Micro RNAs in Wuzhishan Pig Skin by RNA-Sequencing

**Figure 2:**
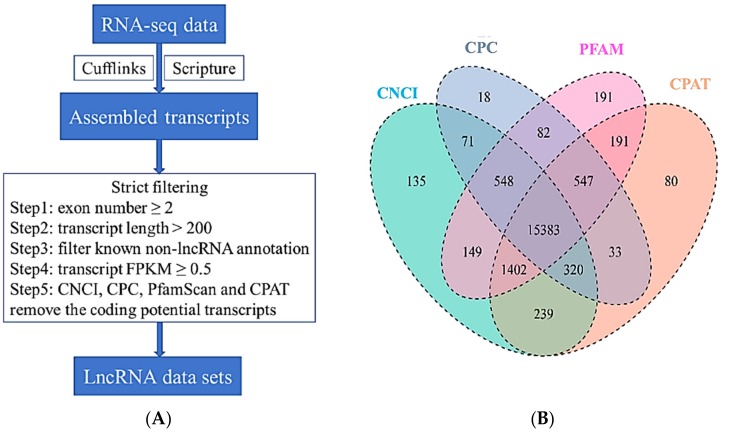
Identification of long non-coding RNAs (lncRNAs) in skin transcriptome. (**A**) Workflow for lncRNA identification. (**B**) Candidate lncRNAs were identified by using four applications: CNCI (coding-non-coding-index), CPC (coding potential calculator), PFAM-scan v1.3 and CPAT (coding potential assessment tool) which detect and remove putative protein-coding transcripts.

**Figure 3:**
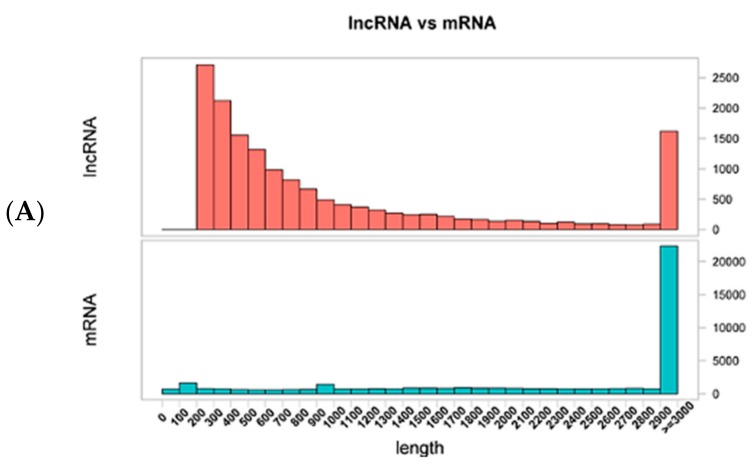

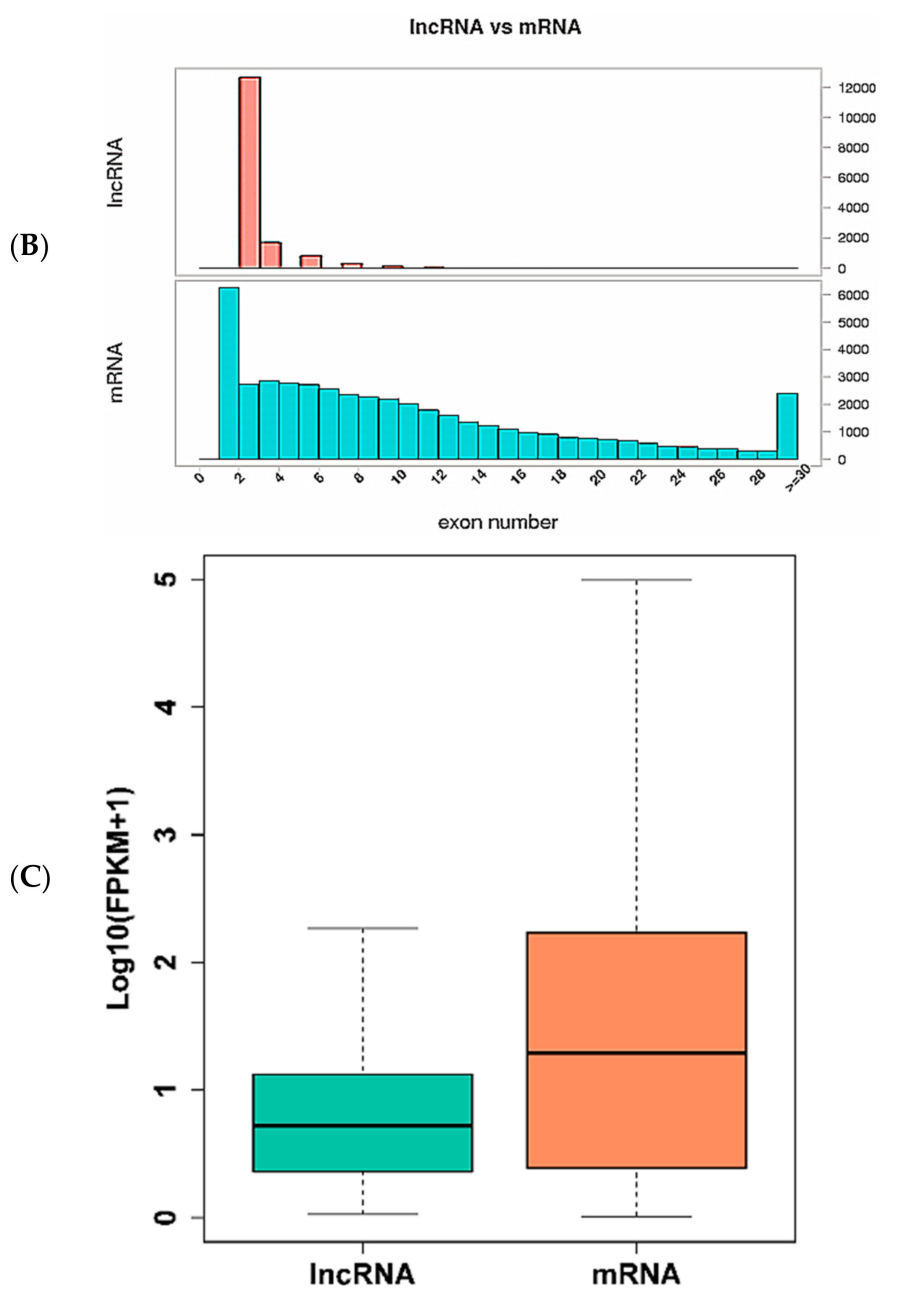
Comparison of genomic architecture and expression level of lncRNAs and messenger RNAs (mRNAs). (**A**) Distribution of lengths of lncRNAs and mRNAs. (**B**) Distribution of number of exons of lncRNAs and mRNAs. (**C**) Expression level of lncRNAs and mRNAs, calculated as log_10_(FPKM + 1). FRKM: Fragments Per Kilobase of exon per Million fragments mapped.

In order to identify lncRNAs, mRNAs and miRNAs involved in coat color, lncRNA and small RNA (miRNA) libraries from black and white skin tissues were constructed using samples obtained from the backs and bellies of three pigs. The lncRNA libraries generated a total of 81–114 million raw reads, of which 79–100 million remained (clean reads) after excluding lower quality data. Approximately 90% of the clean reads were mapped to the reference genome (Table 2) and the correlation coefficients of gene expressions for the three biological replicates were in the range of 0.923–0.976 (Figure S2). After additional filtering (Figure 2A) and removal of potential coding transcripts that were identified using CNCI, CPC, PFAM and CPAT (Figure 2B), a total of 15,383 lncRNAs and 22,990 mRNAs were obtained from the skin samples. The lncRNAs and protein coding genes were compared with respect to gene structure, expression and sequence conservation. lncRNAs were typically shorter than mRNAs (Figure 3A) and tended to contain only two or three exons in contrast to the mRNAs (Figure 3B). lncRNAs also appeared to be expressed at lower levels than mRNAs (Figure 3C). The sequence conservation of lncRNAs was lower than mRNAs (Figure S3).

The miRNA libraries generated a total of 32,232,330 raw reads. A total of 28,431,500 clean reads were retained for further analysis after strict filtering. Most reads were 18–24 nt in length. A total of 55.78% of the small RNAs (sRNAs) were 22 nt long, which was the expected size for miRNAs. A total of 21,750,328 (76.50%) of the clean reads were aligned to the porcine reference sequence. A total of 378 annotated mature miRNAs were identified from 294 precursors (Table S2) while 64 novel mature miRNAs and 69 miRNA precursors were identified (Table S3).

### 3.2. Differential Expression of Genes and Non-Coding RNAs (LncRNA and miRNA) in Skin Tissues

**Figure 4:**
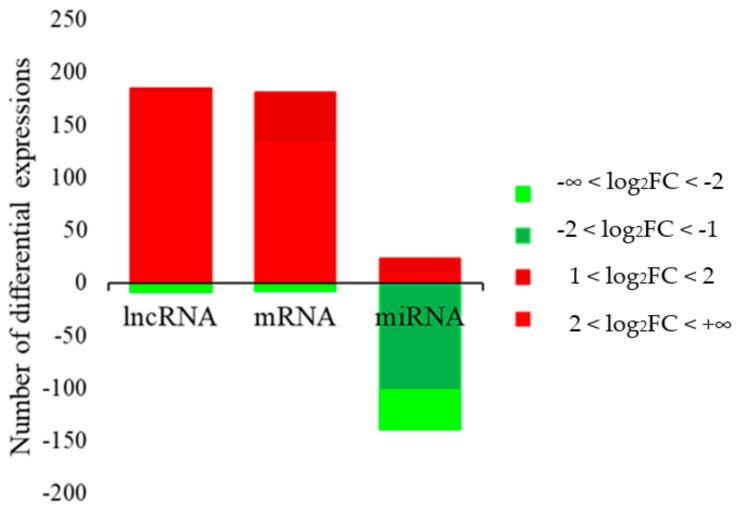
Differentially expressed lncRNAs, mRNAs and miRNAs in skin. Differential expression is shown as log_2_ fold change (FC). RNAs that are relatively more abundant in white skin are shown using red and those that are relatively less abundant are shown in green. Color intensity represents FC magnitude.

We compared gene expression in black skin (from the back) and white skin (from the belly) of Wuzhishan pigs. A total of 189 DEGs were identified (Table S4), among which 181 genes had higher levels of expression and eight genes had lower levels of expression in white skin than in black skin (Figure 4). The largest difference was exhibited by *TYRP1*, which was expressed 30-fold less in white than in black skin (log_2_ fold change = –4.9). Previous studies have shown that *TYRP1* is important for melanogenesis [44,45]. 

Two annotated lncRNAs and 192 novel lncRNAs had significantly different levels of expression in white and black skin (Table S5). In white skin, there were 185 lncRNAs expressed at higher levels and nine expressed at lower levels than in black skin (Figure 4). Several DE lncRNAs were specifically expressed in white skin, such as MSTRG.582214, MSTRG.136277 and MSTRG.548108, or in black skin, such as MSTRG.203809, MSTRG.935672 and MSTRG.872146. These lncRNAs may regulate the synthesis and secretion of melanin.

A total of 136 known miRNAs and 26 novel miRNAs had significantly different levels of expression in white and black skin (Table S6). A total of 23 miRNAs (7 known and 16 novel) were expressed at higher levels and 139 miRNAs (129 known and 10 novel) were expressed at lower levels (Figure 4) in white skin compared to black skin. Among these differentially expressed miRNAs (DE miRNAs), miR-125b [46,47], miR-340 [48,49], miR-21 [50] and miR-145-5p [51] have been reported to be involved in melanogenesis.

### 3.3. Functional Analysis of Differentially Expressed Transcripts

To evaluate the potential functions of differentially expressed genes, lncRNAs and miRNAs, GO and KEGG pathway analyses were performed. GO terms for DEGs were classified into three functional categories (biological process, cellular component and molecular function), which mainly included the cellular process, single-organism process, regulation of biological process, cell part, cell, intracellular and binding. Moreover, the biological processes related to skin or hair follicle pigmentation were significantly enriched, such as melanin biosynthetic process, pigmentation and tyrosine metabolism (Table 3). Pathways related to coat color (“melanogenesis” and “tyrosine metabolism”) were also enriched significantly (Table 3). Several key genes in the melanogenesis pathway were also involved in the synthesis of melanin, such as *TYR*, *TYRP1*, *DCT* and *CAMK2A*.

Long non-coding RNAs can regulate protein-coding gene expression at both the transcriptional and post-transcriptional levels [52]. To identify genes that are potentially regulated by lncRNAs, genes located 50 kb upstream and downstream from lncRNA loci were examined as potential cis targets. The genes with specific Pearson correlations (*r* > 0.90 or *r* < –0.90) were selected as the potential trans-targets. A total of 8241 genes were predicted to be targets of 194 DE lncRNAs, including 195 cis and 8107 trans target genes (61 were shared). GO analysis suggested that cis and trans target genes are mostly involved in the regulation of biological, metabolic and cellular processes (Figure S4A). KEGG pathway analysis indicated that the genes targeted by DE lncRNAs were involved in pathways related to endocytosis, AMPK signaling and metabolism (Figure S4B). In addition, our further analysis showed that 61 cis-trans-shared genes were mainly involved in cellular process, binding, single-organism process, endocytosis and metabolic pathways.

To examine the potential biological functions of the DE miRNAs, we used miRanda to predict target genes. A total of 21,068 target genes were identified, of which 15,009 and 6059 genes were predicted to be targets of 136 known miRNAs and 26 novel miRNAs, respectively. GO analysis suggests that DE miRNAs target the genes that are predominantly involved in biological processes, such as cellular processes, metabolic processes, biological regulation and developmental processes (Figure S4C). The MAPK signaling pathway, melanogenesis and Wnt signaling pathway were significantly enriched in the KEGG analysis (Figure S4D).

### 3.4. LncRNA–miRNA–Gene Interaction Network Construction

**Figure 5:**
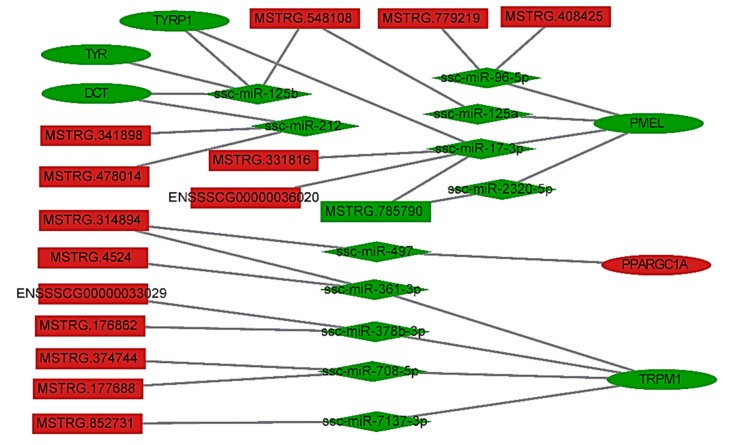
LncRNA–miRNA–gene interaction network. Rectangles, diamonds and ovals represent lncRNAs, miRNAs and genes, respectively. Red indicates that the RNA is relatively more abundant, and green indicates that the RNA is relatively less abundant in white skin than in black skin.

Recently, lncRNAs and mRNAs have been demonstrated to function as ceRNAs in diverse physiological and pathophysiological states through their ability to bind miRNAs at sites known as microRNA response elements (MREs) [53,54,55]. Therefore, we used miRanda and TargetScan (http://www.targetscan.org/) [56] to screen lncRNAs and genes for MRE sequence motifs and then constructed a lncRNA–miRNA–gene interaction network (Figure 5) to examine the relationships between 194 DE lncRNAs, 163 DE miRNAs and 10 DEGs (Table 3). The network shows possible interactions amongst 12 lncRNAs, 23 miRNAs and 7 genes. For example, miR-125b potentially binds MSTRG.548108 and the *TYR*, *TYRP1* and *DCT* mRNAs while ssc-miR-17-3p may bind MSTRG.331816, MSTRG.785790, ENSSSCG00000036020 and the *PMEL* and *TYRP1* mRNAs. These results suggest that genes, lncRNAs and miRNAs that are differentially expressed in white and black skin may cooperatively regulate skin physiology and melanogenesis via an interaction network.

### 3.5. Quantitative Polymerase Chain Reaction Validation of Differentially Expressed Transcripts

**Figure 6:**
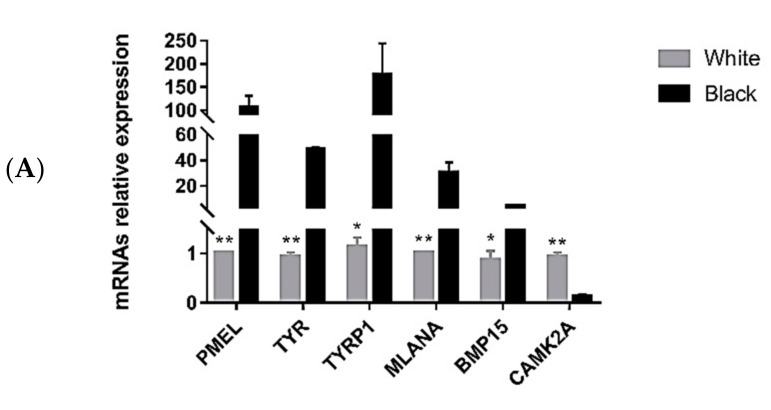

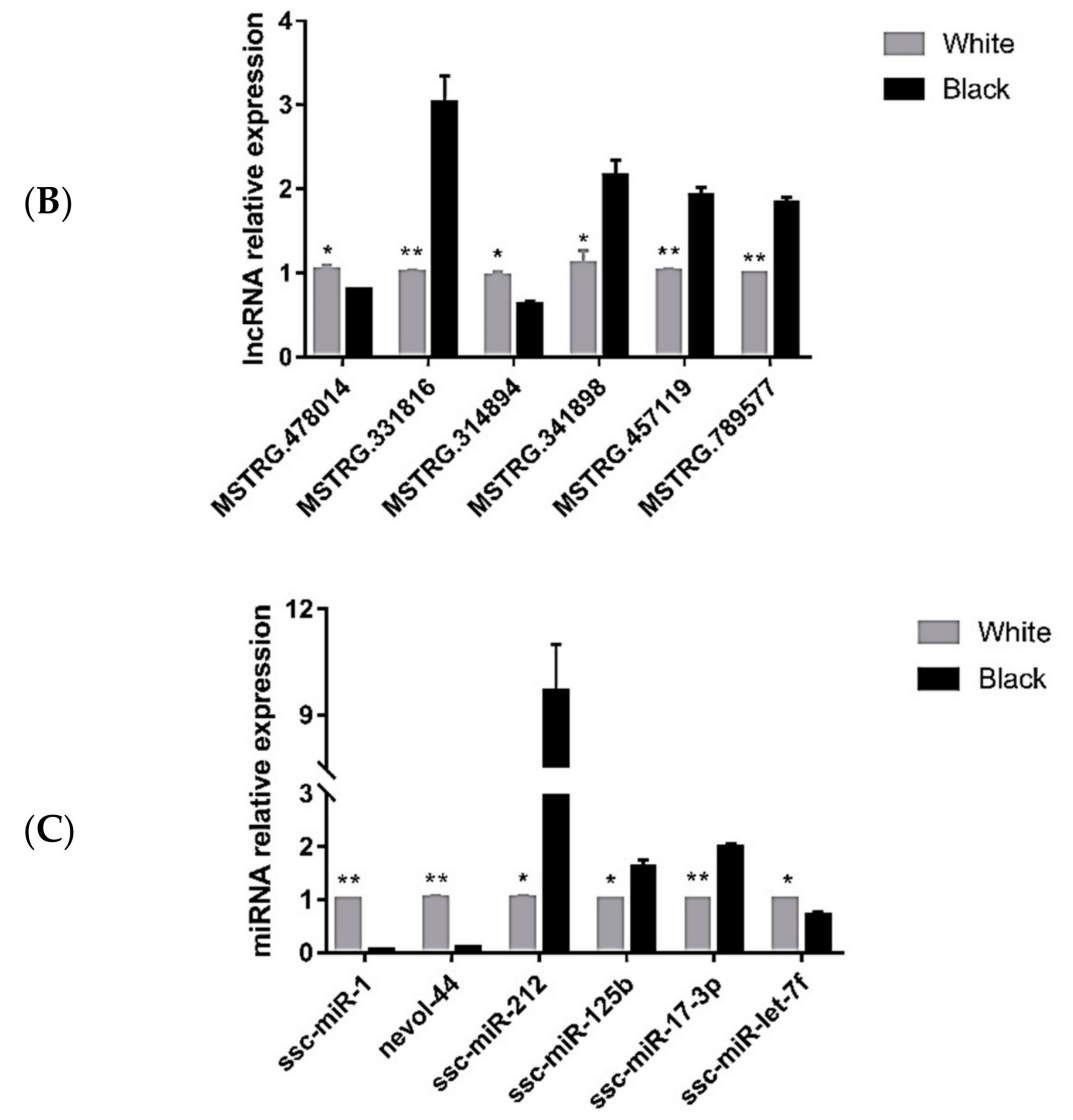
Quantitative PCR validation. Differentially expressed genes (**A**), lncRNAs (**B**) and miRNAs (**C**) were confirmed by quantitative PCR. Results are shown as means ± standard deviation of triplicate measurements. * indicates *p* < 0.05, ** indicates *p* < 0.01.

To validate the RNA-seq results, six lncRNAs, six mRNAs and six microRNAs were selected and their expression patterns in black and white skin were examined using qPCR. Expression patterns were consistent with expression levels calculated from the RNA-seq data (Figure 6A–C). 

## 4. Discussion

The darker pigmentation of hair follicles and skin was associated with a higher number of melanosomes, although the number of melanocytes remains constant [57]. Melanocytes in black skin or hair follicles contain the highest number of melanosomes, whereas melanocytes in white skin or hair bulbs contain the lowest [58]. The relationship between the lower levels of melanin and a lighter coat color has been observed in various species, including mice [59], horses [60], sheep [61], alpacas [62] and humans [63]. In Wuzhishan pigs, two mechanisms have been proposed for the black back and white belly coat color pattern. Based on the above research, one hypothesis states that the phenotype is caused by a difference in the number of melanosomes, with the black back having more melanin and the white abdomen having less. An alternative hypothesis proposes that the difference reflects a mechanism involving epigenetic regulation, such as non-coding RNA [64]. Because high throughput RNA sequencing technology has made it possible to identify key genes associated with specific phenotypes or important biological processes, it may be possible to decipher the mechanisms that underlie coat color patterns. RNA sequencing has been used to study the skin transcription profile associated with coat color in a number of animals, including sheep [10], horses [14], chickens [21] and mink [13]. Among these studies, common genes (such as *DCT*, *TYR* and *TYRP1*) and pathways (melanogenesis) were identified, which participate in the regulation of coat color. Recent RNA-seq studies have shown that transcriptional changes in the skin are associated with a piebald phenotype in cattle [26], but similar studies have not been conducted in pigs. 

In this study, RNA-seq was used to examine the patterns of transcription in skin obtained from the back (black) and belly (white) of individual Wuzhishan pigs. A large number of differentially expressed transcripts were detected, including DE lncRNAs, DEGs and DE miRNAs. Amongst the DEGs, several (Table 3) were associated with pigmentation of the skin and hair follicle by functional analysis. These genes were significantly enriched in the melanogenesis, tyrosine metabolism and Wnt signaling pathways, which suggests that they are involved in the formation of pigmentation. The premelanosome protein *PMEL* (also known as *SILV* or *PMEL17*), a key component of mammalian melanosome biogenesis, is required for the generation of cylindrical melanosomes in zebrafish, which in turn are required for melanosome movement into the apical processes [65]. Mutations in *PMEL* have previously been shown to affect hypopigmented phenotypes in many vertebrates, such as mice [66], chickens [67] and dogs [68]. Using quantitative PCR, we found a higher level of *PMEL* expression in back skin with a black coat color. These results suggest that a lower level of *PMEL* expression is responsible for a deficiency in the biosynthesis of melanin (hence the white coat color in abdominal skin) and may be the direct cause of the back black and belly white coat color in the same individual. This simple model is also consistent with studies in mink, where *PMEL* showed a higher level of expression in black mink skin [13]. 

*MLANA* (*MART-1*) is localized in melanosomes [69]. *MART-1* interacts with *PMEL17* and plays an important role in regulating mammalian pigmentation [70]. *MART-1*, together with ocular albinism type 1(*OA1*), controls melanosome identity and composition in the early stages of melanogenesis [71]. Moreover, *MART-1* is highly enriched in early melanosomes (Stage I and/or II melanosomes). Using quantitative PCR, we detected lower levels of *MLANA* in skin from the abdomen (white) and a higher level of expression in skin from the back (black). These results suggest that *MLANA* has an important role in early melanogenesis and is responsible for the different coat colors in the same individual.

*TRPM1* expression positively correlates with melanin content in melanocytes [72]. Because *TRPM1* is a Ca^2+^ permeable ion channel, its activation increases intracellular Ca^2+^ levels, which results in an increase in melanin production. Indeed, the knockdown of *TRPM1* in melanocytes reduces intracellular Ca^2+^ and decreases tyrosinase activity and melanin pigmentation [73]. We found that *TRPM1* is expressed at different levels (log_2_ fold change = −2.46) in black and white skin. This result suggests that *TRPM1* might contribute to coat color variations in the same individual by affecting melanin content in melanocytes.

Non-coding RNAs (ncRNAs) have evolved in eukaryotes as epigenetic regulators of gene expression. miRNAs and lncRNAs are the most abundant regulatory ncRNAs. Each ncRNA class regulates gene expression through distinct mechanisms. Many miRNAs play crucial roles in melanin regulation during melanogenesis. For example, miR-340 participates in the formation of pigmentation by regulating *MITF* in melanocytes [49] and is involved in regulating UVB-induced dendrite formation and the aggregation of melanosomes in the dendritic tips of human melanocytes [48]. The overexpression of miR-145 leads to hypopigmentation as it targets genes implicated in the first step of melanogenesis in mouse melanocytes (*SOX9*, *MITF*, *TYR* and *TYRP1*) and genes involved in melanosome transport (*MYO5A* and *RAB27A*) in human melanocytes [51]. The same study identified other potential regulatory miRNAs, such as miR-125b, miR-145, miR-221, miR-206 and miR-222, which are consistent with our results (Table S6). We also identified 26 novel DE miRNAs, which suggests that there may be even more miRNAs associated with melanogenesis to be discovered. Taken together, the results suggest that these DE miRNAs are important in the formation of the black back and white belly coat color phenotype in Wuzhishan pigs.

In contrast to miRNAs, functional analysis demonstrated that genes targeted by DE lncRNAs were not enriched in pathways related to melanogenesis, tyrosine metabolism or Wnt signaling. We speculate that lncRNA may regulate coat color indirectly by ceRNA rather than directly by acting on target genes. Although a few published reports have examined the role of lncRNAs in melanomas, it is not known if lncRNAs are involved in the complex mechanisms underlying melanogenesis and pig coat color. Therefore, we constructed the lncRNA–miRNA–gene interaction network (Figure 5). Our analysis suggests that the MSTRG.548108-miR-125b pair interacts with genes, including *TYR*, *TYRP1* and *DCT*. miR-125b is a known regulator of melanogenesis. The upregulation of miR-125b using miR-125b mimics reduces the expression of pigmentation-related genes (*TYR*, *TYRP1* and *DCT*) as well as the melanin content in human primary melanocytes and human pigmented tissues [46]. The same authors identified SRC homology 3 domain-binding protein 4 (*SH3BP4*) as a pigmentation-related gene regulated by miR-125b [47]. Interestingly, *TYR*, *TYRP1* and *DCT* are directly involved in the synthesis of melanin. Moreover, MSTRG.548108 showed white-specific expression. These results strongly suggest that MSTRG.548108 is involved in melanogenesis through the interaction networks involving MSTRG.548108-miR-125b with *TYR* or *TYRP1* or *DCT*, which results in the black and white coat color of Wuzhishan pigs. lncRNAs expand the list of mechanisms that potentially orchestrate the genetic regulation of melanogenesis. We anticipate that the discovery of new roles for lncRNAs and the discovery of new lncRNAs will advance our understanding of the coat color phenotype in the pig.

Taken together, our data provide evidence for interactions that have high functional specificity in melanogenesis and that are consistent with the ceRNA hypothesis. This study provides new insights into the complex molecular mechanisms underpinning pig coat color variation but understanding exactly how the different coat colors arise in the back and belly of Wuzhishan pigs will require additional experiments. 

## 5. Conclusions

In this study, we compared the expression of genes, lncRNAs and miRNAs in the white and black skin tissues from black-back/white-belly Wuzhishan pigs. Our analysis suggests that several genes, lncRNAs and miRNAs are involved in important biological processes associated with melanogenesis. We have also constructed the first lncRNA–miRNA–gene interaction networks based on the transcription profiles derived from pig skin. The study contributes to our understanding of the complex molecular mechanisms underlying skin physiology and melanogenesis in the pig in addition to expanding our knowledge of the origin of coat color diversity in domestic animals.

## Figures and Tables

**Table 1:** Differentially expressed lncRNAs, genes and miRNAs in white vs. black skin from black-back/white-belly Wuzhishan pigs.

Genes	Gene ID	Gene Name	White vs. Black log_2_FC	*p*-Value
lncRNAs	MSTRG.789577		–5.51	0.0019
	MSTRG.478014		2.20	0.0346
	MSTRG.331816		2.92	0.0317
	MSTRG.314894		2.71	0.0236
	MSTRG.341898		3.35	1.09E–04
	MSTRG.457119		5.82	1.89E–06
genes	ENSSSCG00000026409	*TYR*	–2.03	0.0428
	ENSSSCG00000005206	*MLANA*	–2.68	0.0487
	ENSSSCG00000000371	*PMEL*	–3.84	5.64E–09
	ENSSSCG00000005193	*TYRP1*	–4.93	1.58E–04
	ENSSSCG00000014443	*CAMK2A*	2.80	5.29E–04
	ENSSSCG00000012310	*BMP15*	8.16	5.46E–12
microRNAs		ssc-miR-125b	–1.76	0.0000
		ssc-miR-17-3p	–3.17	0.0057
		ssc-miR-212	–4.76	1.14E–04
		ssc-let-7f	1.47	0.0000
		ssc-miR-1	2.48	0.0000
		Novel-44	15.37	0.0000

FC: Fold change.

**Table 2:** Statistical summary for RNA sequencing results.

Sample Name	Raw Reads	Clean Reads	Error Rate (%)	Q20 (%)	Q30 (%)	% of Mapped Reads	Uniquely Mapped Reads
WW1	97269174	85519188	0.02	96.78	92.01	88.89	74229023
WW2	102600722	83351954	0.02	96.94	92.43	90.72	73653848
WW3	84105516	82637176	0.02	97.14	92.52	95.13	76182061
WB1	98557820	81182410	0.02	96.70	91.90	90.64	71722643
WB2	113729814	100498792	0.02	96.70	91.89	90.78	89001334
WB3	81046142	79,101,022	0.02	97.48	92.66	95.09	72977205

**Table 3:** Significantly enriched GO terms and KEGG pathways related to skin or hair follicle pigmentation. Bold italic and italic text respectively indicate higher and lower expression of differentially expressed genes (DEGs) in white skin compared to black skin.

Category	Terms	DEGs No.	*p*-Value	Genes
GO	GO:0042438-melanin biosynthetic process	4	1.38E–05	*PMEL*, *TYR*, *TYRP1*, *DCT*
	GO:0048066-developmental pigmentation	4	4.7E–04	*PMEL*, *TYR*, *TYRP1*, *DCT*
	GO:0006582-melanin metabolic process	4	1.66E–05	*PMEL*, *TYR*, *TYRP1*, *DCT*
	GO:0043473-pigmentation	4	0.00456	*PMEL*, *TYR*, *TYRP1*, *DCT*
	GO:0042440-pigment metabolic process	5	1.1E–04	*PMEL*, *TYR*, *TYRP1*,***PPARGC1A***, *DCT*
	GO:0033162-melanosome membrane	3	6.52E–05	*TYRP1*, *TYR*, *DCT*
	GO:0006570-tyrosine metabolic process	2	0.00243	*TYR*, *DCT*
	GO:0042470-melanosome	5	8.7E–04	*PMEL*, *MLANA*, *TYR*, *TYRP1*, *DCT*
	GO:0048770-pigment granule	6	8.7E–04	*PMEL*, *MLANA*, *TYR*, *TYRP1*, *DCT*, *TRPM1*
	GO:0046148-pigment biosynthetic process	4	5.0E–04	*PMEL*, *TYR*, *TYRP1*, *DCT*
KEGG pathway	hsa00350-Tyrosine metabolism	3	0.0022	*TYRP1*, *TYR*, *DCT*
	hsa04916-Melanogenesis	4	0.0056	*TYRP1*, *TYR*, *DCT*, ***CAMK2A***

## Supplementary Materials

The following are available online at https://www.mdpi.com/2073-4425/10/3/201/s1. Figure S1: Primers used to detect gene and lncRNA expression Table S2: Annotated mature miRNAs and precursors, Table S3: Novel mature miRNA sequences and precursor sequences, Table S4: Differentially expressed genes in white skin and black skin, Table S5: Differentially expressed lncRNAs in white skin and black skin, Table S6: Differentially expressed miRNAs in white skin and black skin, Figure S1: Dopa staining of black skin and white skin of Wuzhishan pig, Figure S2: Pearson correlation coefficients for the biological replicates from WW (white skin) and WB (black skin), Figure S3: The sequence conservation of lncRNAs and mRNAs, Figure S4: Crucial pathways were clustered from DE lncRNAs and DE miRNAs target genes.
