# Supplementary material for: Transcriptomic Analysis of Coding Genes and Non-Coding RNAs Reveals Complex Regulatory Networks Underlying the Black Back and White Belly Coat Phenotype in Chinese Wuzhishan Pigs
Source: Genes (Basel). 2019 Mar 7;10(3):201. doi: 10.3390/genes10030201 (PMC6470719; doi:10.3390/genes10030201)
Supplement: Supplementary file 1 [file genes-10-00201-s001.zip › Figure S1 Dopa staining of black skin and white skin of Wuzhishan pig.docx]

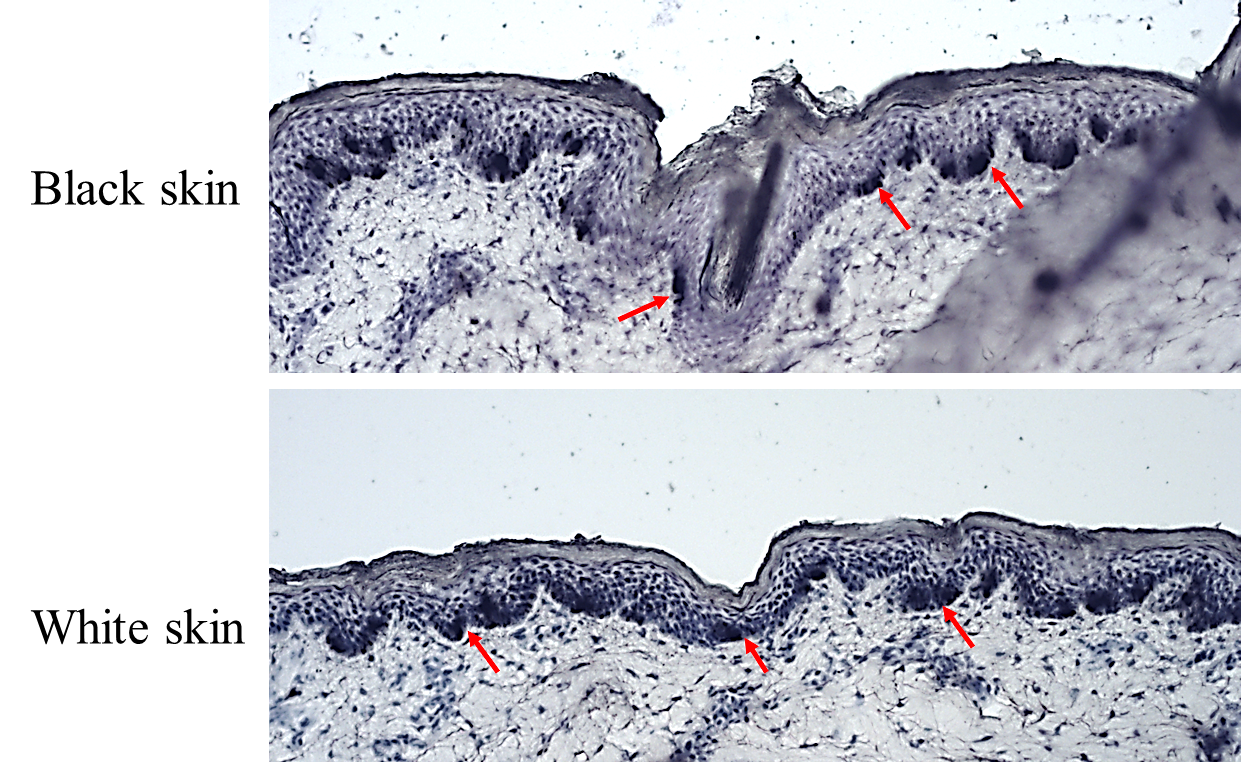


**Figure S1.** Dopa staining of black skin and white skin of Wuzhishan pig. The red arrow indicates melanocytes.
