# Supplementary material for: Transcriptomic Analysis of Coding Genes and Non-Coding RNAs Reveals Complex Regulatory Networks Underlying the Black Back and White Belly Coat Phenotype in Chinese Wuzhishan Pigs
Source: Genes (Basel). 2019 Mar 7;10(3):201. doi: 10.3390/genes10030201 (PMC6470719; doi:10.3390/genes10030201)
Supplement: Supplementary file 1 [file genes-10-00201-s001.zip › Figure S4 Crucial pathways were clustered from DE lncRNAs and DE miRNAs target genes.docx]

**
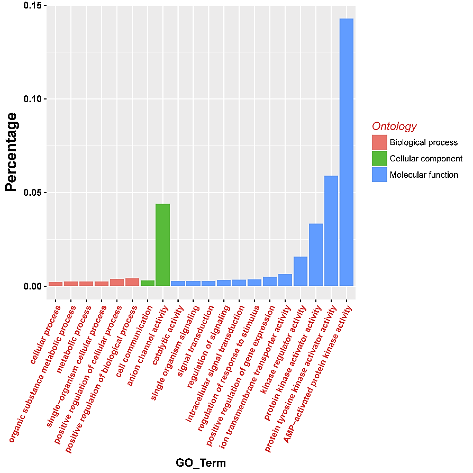
A**

**
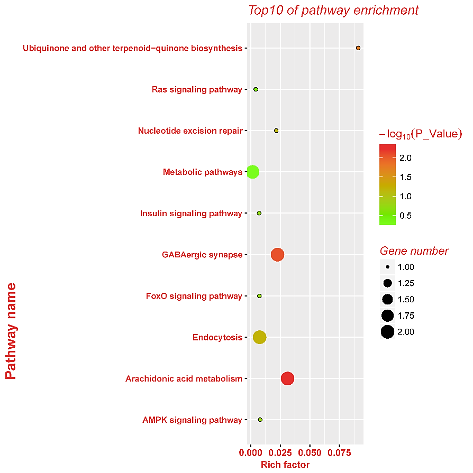
B**

**
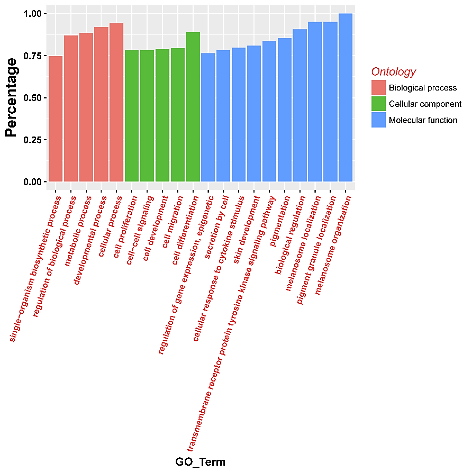
C**

**
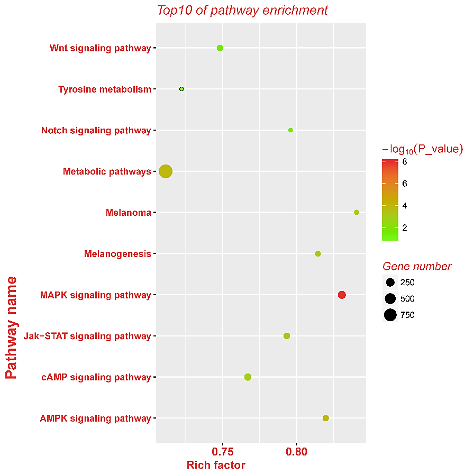
D**

(**d**)

**Figure S2.** Crucial pathways were clustered from DE lncRNAs and DE miRNAs target genes. GO terms analysis (**A**, **C**) and KEGG pathways (**B**, **D**) clustered by DE lncRNAs and DE miRNAs, respectively.
