# Supplementary material for: Transcriptomic Analysis of Coding Genes and Non-Coding RNAs Reveals Complex Regulatory Networks Underlying the Black Back and White Belly Coat Phenotype in Chinese Wuzhishan Pigs
Source: Genes (Basel). 2019 Mar 7;10(3):201. doi: 10.3390/genes10030201 (PMC6470719; doi:10.3390/genes10030201)
Supplement: Supplementary file 1 [file genes-10-00201-s001.zip › Table S1 Primers used to detect gene and lncRNA expression.docx]

**Table S1.** Primers used to detect gene and lncRNA expression.

| Genes | Gene name/ID | | Primers | E (%) | Amplicon Length(bp) |
| --- | --- | --- | --- | --- | --- |
| gene | | *TYR* | F: 5' GCACAGAGAGGCGACTTTTG 3'  R: 5' GGGCTGGTAGTATGTTTCGC 3' | 103 | 106 |
|  |  | *MLANA* | F: 5' GGGCATTTTACTTCTCATCAGC 3'  R: 5' CACAGGTCCACAGTTGTTCTCC 3' | 97 | 178 |
|  |  | *PMEL* | F: 5' GGGACCTACTGCCTCAATG 3'  R: 5' CCAACAAGATGCCCACGAA 3' | 95 | 127 |
|  |  | *TYRP1* | F: 5' GGAGAAACCTACTGGACCTAAG 3'  R: 5' ATCTCCTGGTGGCAATGACG 3' | 95 | 108 |
|  |  | *CAMK2A* | F: 5' CCAGTTCCAGCGTTCAGTTA 3'  R: 5' CTTCGTGTAGGACTCAAAATCT 3' | 98 | 152 |
|  |  | *BMP15* | F: 5' CCCTCGGGTACTACACTATG 3'  R: 5' GGCTGGGCAATCATATCCT 3' | 98 | 192 |
| lncRNA | | MSTRG.789577 | F: 5' CAGAGCGGGACTGAACCTTG 3'  R: 5'CCAGTGGCTACAGTTCCGAT 3' | 90 | 211 |
|  |  | MSTRG.478014 | F: 5' CACAGCAAATCACTGCCAACT 3'  R: 5' CACAAAGCATCATCATAACCAA 3' | 87 | 248 |
|  |  | MSTRG.331816 | F: 5' AGGCTGAAAAGGGAGGCA 3'  R: 5' GCTTCTGAGTCTTCCATCTTGC 3' | 101 | 208 |
|  |  | MSTRG.314894 | F: 5' AAGGCAGAGCAAAGGGTAGA 3'  R: 5' ACTACTTCACTTGGATGGGTTT 3' | 87 | 119 |
|  |  | MSTRG.341898 | F: 5' AGGGAGGCAGAACAGTGTATGG 3'  R: 5' ACTTGGCATTTCCTGGTTCC 3' | 96 | 136 |
|  |  | MSTRG.457119 | F: 5' CCCCTTCGGCAATCATCAT 3'R: 5' AGGAGAAGTGAGGTGCTGGC 3' | 101 | 172 |
