# Supplementary figures and images for: Transcriptomic Analysis of Coding Genes and Non-Coding RNAs Reveals Complex Regulatory Networks Underlying the Black Back and White Belly Coat Phenotype in Chinese Wuzhishan Pigs
Source: Genes (Basel). 2019 Mar 7;10(3):201. doi: 10.3390/genes10030201 (PMC6470719; doi:10.3390/genes10030201)

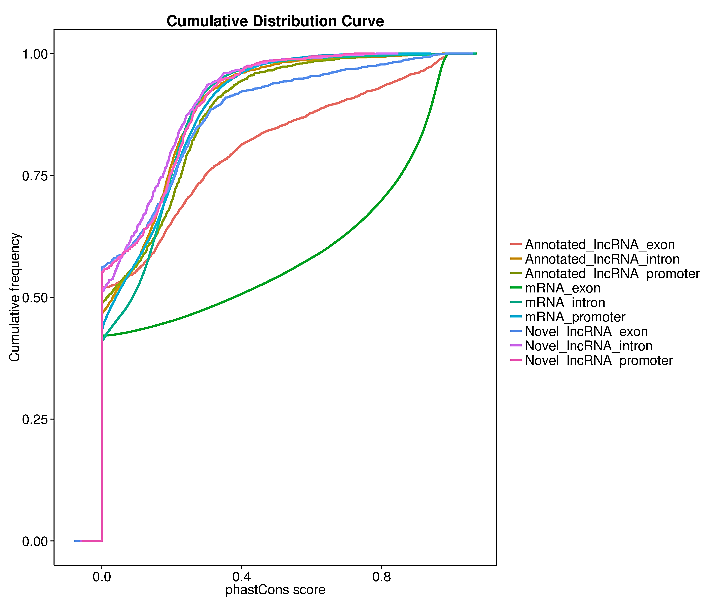


**Figure S2.** The sequence conservation of LncRNAs and mRNAs.

Supplement: Supplementary file 1 [file genes-10-00201-s001.zip › Figure S3 The sequence conservation of LncRNAs and mRNAs.docx]
